# Clinical characteristics, diagnosis, treatment, and prognostic factors of pulmonary mucosa‐associated lymphoid tissue‐derived lymphoma

**DOI:** 10.1002/cam4.2683

**Published:** 2019-11-05

**Authors:** Lin Wang, Guanzhi Ye, Zhonghe Liu, Lin Shi, Cheng Zhan, Jie Gu, Rongkui Luo, Zongwu Lin, Di Ge, Qun Wang

**Affiliations:** ^1^ Department of Thoracic Surgery Zhongshan Hospital Fudan University Shanghai China; ^2^ Department of Pulmonary Medicine Zhongshan Hospital Fudan University Shanghai China; ^3^ Department of Pathology Zhongshan Hospital Fudan University Shanghai China

**Keywords:** chemotherapy, MALT lymphoma, prognosis, pulmonary lymphoma, thoracic surgery

## Abstract

Primary pulmonary mucosa‐associated lymphoid tissue‐derived (MALT) lymphoma is a rare disease with a favorable prognosis. However, its clinical characteristics, diagnosis, treatment, and prognoses remain unclear. We retrospectively analyzed 80 patients with pathologically confirmed MALT lymphoma from 2006 to 2018. The clinical characteristics, diagnosis, treatments, and prognoses of all the 80 patients were recorded. Patients were stratified into surgery and biopsy groups, respectively, to evaluate the role of surgery in the diagnosis and treatment of MALT lymphoma. The prognoses were compared between different clinical characteristics and treatments. Pathological diagnoses were confirmed by surgery, bronchoscopy, and percutaneous biopsy. Thirty patients were treated by surgery. While MALT lymphoma was only diagnosed by bronchofiberoscopy or bercutaneous biopsy in four of 18 patients in the surgery group who underwent the procedure. Six patients received adjuvant chemotherapy and one patient received involved‐field radiotherapy in surgery group. Thirty‐one patients were treated with chemotherapy alone, one patient was treated with radiotherapy, one patient received only symptomatic and supportive treatment, and waiting and watching without treatment were recommended in 17 patients in biopsy group. Eight patients died during follow‐up and the 5‐year survival rate was 87.1%. Tumor number, treatment, and age were prognostic factors for overall survival (OS), but age was the only independent prognostic factor according to multivariate analysis. While, tumor number was the only prognostic factor in the analysis about progression‐free survival (PFS). No significant difference was found in OS or PFS between patients treated with and without surgical resection. MALT lymphoma is an indolent disease with favorable treatment outcome. Tumor number is associated with PFS and age is the only significant prognostic factor for pulmonary MALT lymphoma patients because of its indolent nature, but surgery still plays an important role in the diagnosis and treatment of MALT lymphoma.

## Graphical abstract

MALT lymphoma is an indolent disease with favorable treatment outcomes. There was no significant difference in overall survival between patients treated with and without surgical resection, but surgery still plays an important role in the diagnosis and treatment of MALT.

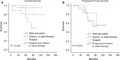

## 1 INTRODUCTION

Primary pulmonary non‐Hodgkin lymphomas are extremely rare, comprising only 0.5%–1.0% of all lung malignancies^1^ and only 0.4% of all lymphomas.^2^ Mucosa‐associated lymphoid tissue‐derived (MALT) lymphoma is the most common type of primary pulmonary lymphoma.^3^

No specific imaging manifestations can be found on computed tomography (CT) images for pulmonary MALT lymphomas and single or multiple areas of consolidation, masses, nodules, and/or other rarer findings may appear on CT images.^4^ The entity may thus be easily misdiagnosed because of its nonspecific clinical symptoms and imaging examination results.

Infection has been suggested as possible cause of MALT lymphoma. The association between gastric MALT lymphoma and *Helicobacter pylori* is a well‐known example.^5^ The association between *B burgdorferi* and cutaneous MALT lymphoma, hepatitis C virus, and hepatic MALT lymphoma, and *C psittaci* and ocular adnexal MALT lymphoma are other suspected links between infection and MALT lymphoma.^6^, ^7^, ^8^, ^9^ Whereas the association between pulmonary MALT lymphoma and chronic pulmonary infection is still suspected but not yet demonstrated. A previous study suggested that the incidence of individual Chlamydiae was generally higher in pulmonary MALT lymphoma than nonlymphoproliferative disorders, but it did not reach statistical significance.^10^ Recently, a European research group examined pulmonary MALT lymphoma cases and reported that DNA from *Achromobacter xylosoxidans* was found in 57 out of 124 pulmonary MALT lymphomas versus only 15 of 82 controls.^11^ However, *A xylosoxidans* DNA was detected in 1/52 cases of pulmonary MALT lymphoma in the study of a Japanese case series.^12^ Therefore, the causative antigen associated with pulmonary MALT lymphoma has not yet been identified. Nonspecific pulmonary symptoms such as cough, and fever were commonly presented in pulmonary MALT lymphoma patients. These symptoms were often associated with pulmonary inflammation, which significantly increased the difficulty of differential diagnosis of pulmonary MALT lymphoma from pulmonary inflammation. Primary pulmonary MALT lymphoma is associated with good survival, with a 5‐year survival rate over 85%.^13^ Treatment options include surgery, radiotherapy, chemotherapy, and observation. Although there are several reports about the treatment of pulmonary MALT lymphoma, the low incidence, slow progression, and good prognoses of various treatment options of pulmonary MALT lymphoma mean no standard therapeutic strategies or guidelines for this disease.

This study retrospectively investigated the characteristics and treatments associated with prognoses in a series of primary pulmonary MALT lymphoma to determine the optimal diagnostic procedures and treatments.

## 2 PATIENTS AND METHODS

The study protocol was approved by the Ethics Committee of Zhongshan Hospital, Fudan University, Shanghai, China. Written informed consent was obtained from all patients for use of surgical samples and clinical information for medical research.

**Figure 1:**
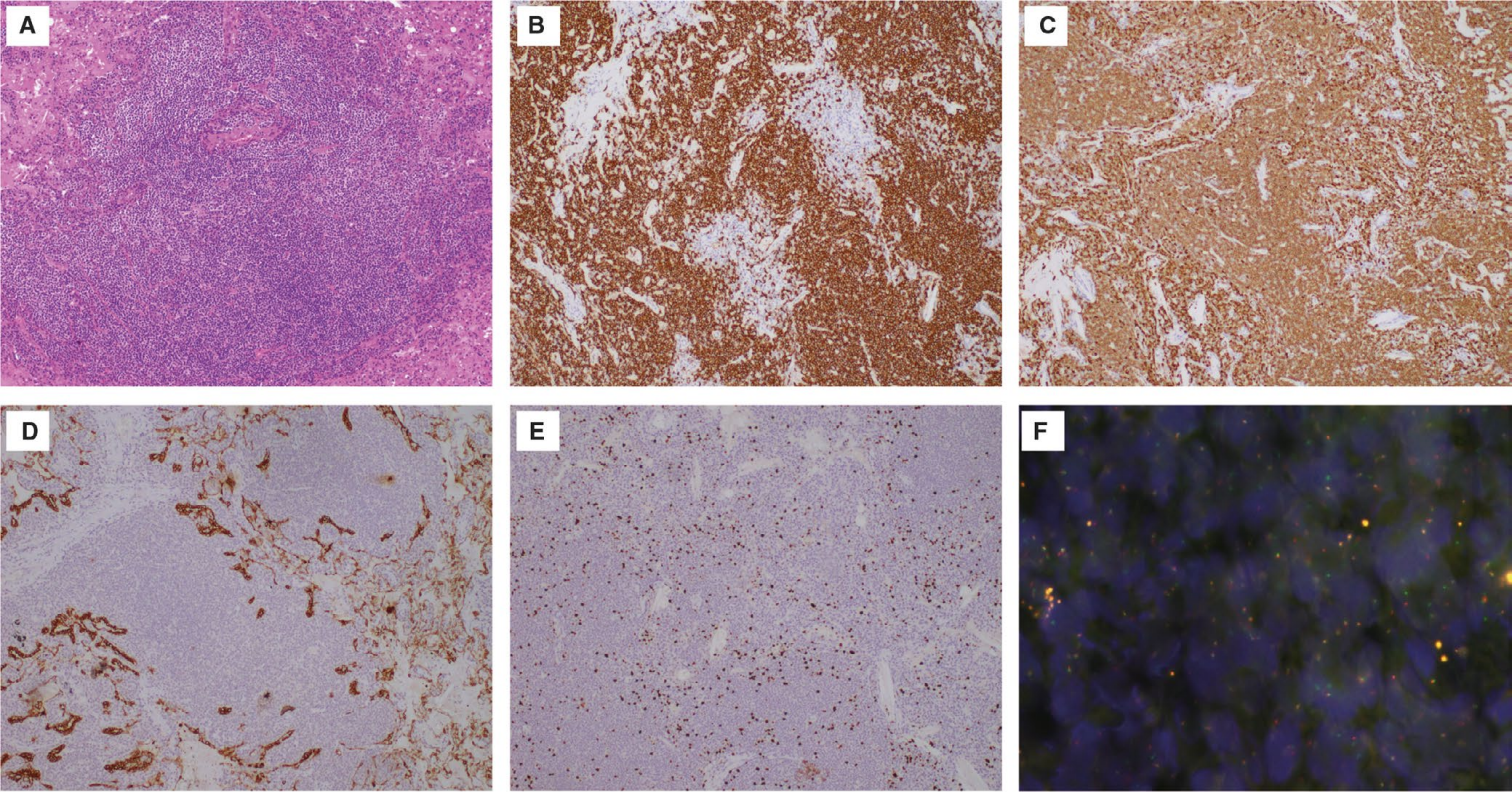
Morphology and immunophenotyping images of tissue from the patients with pulmonary MalT lymphoma. A, Abnormal lymphocyte infiltration, HE staining, original magnification × 100; B‐E, Immunohistochemical staining for CD20 (B); CD79a (C); CK7 (D); and Ki‐67 (E) expression. Original magnification 100×. F, MALT‐1 gene rearrangements using FISH

All patients diagnosed with pulmonary MALT lymphoma at Zhongshan Hospital, Fudan University, from January 2006 to December 2018 were retrospectively evaluated. Eligible patients were pathologically diagnosed with pulmonary MALT lymphoma and had complete medical information, including clinical characteristics and prognoses. Diagnosis was obtained by surgical resection, surgical biopsy, transbronchial lung biopsy, or CT‐guided transthoracic lung biopsy. The diagnosis of MALT lymphoma was according to World Health Organization criteria.^14^ Morphologically, the tumor cells have small to medium‐sized lymphocytes, slightly irregular nuclei with inconspicuous nucleoli, resembling those of centrocytes, and relatively abundant, pale cytoplasm. Immunohistochemical staining was positive for CD20 and CD79a expression. In cases in which it proved difficult to exclude other low‐grade B‐cell lymphomas, immunohistochemical studies for CD5, CD10, CD23, cyclin D1, and Ki‐67 were conducted. The detection of MALT‐1 gene rearrangements using fluorescence in situ hybridization (FISH) techniques was also used in the pathological diagnosis. Because MALT lymphoma is commonly accompanied by reactive hyperplasia, gene rearrangement studies for the IgH gene were conducted via PCR analysis, in an attempt to exclude any benign hyperplasia. Meanwhile, the primary site of MALT lymphoma was in the lungs, either with single or multifocal lesions (including cases with bilateral presentation), with or without lymph node involvement. Examples of representative pathologic images of tissue from patients in this study are shown in Figure 1.

Staging was performed according to the modified Ann Arbor staging.^2^ Initial pulmonary MALT lymphoma staging was on the basis of a detailed medical history, physical examination, and radiographic examination, including chest CT and positron emission tomography/CT. The number of lung lesions on chest CT were recorded. Patients were treated with chemotherapy, radiotherapy, simple follow‐up, surgical resection, or surgery plus adjuvant treatment, according to the imaging findings and stage.

Progression‐free survival (PFS) and overall survival (OS) were compared using the Kaplan‐Meier method with log‐rank test. PFS was defined as the time from date of diagnosis until progression. OS was defined as the time between the date of diagnosis and the date of death or the last follow‐up visit. Multivariate analysis and Cox proportional hazards regression analysis were conducted including variables that achieved statistical significance in univariate analysis, to identify factors independently associated with survival. Statistical analyses were performed using IBM SPSS Statistics for Windows, version 22.0 (IBM Corp.). A two‐tailed value of *P* less than .05 was considered statistically significant.

## 3 RESULTS

The baseline characteristics of enrolled patients are summarized in Table 1. This study included a total of 80 patients, including 37 men and 43 women, with a median age of 59 years (range: 31‐80 years). Twenty‐seven patients exhibited no symptoms, whereas respiratory symptoms including cough, dyspnea, sputum, and chest pain were the most common symptoms among the other patients, occurring in 66.3% of the total population. B symptoms were observed in 17 patients. Thirty‐six patients had single lesions, 11 had two lesions, and 33 patients had three or more lesions. Pathological diagnoses were confirmed by surgery, bronchoscopy, and percutaneous biopsy. Pathological specimens were obtained by biopsy in 18 patients in the surgery group, but only four of these resulted in a definitive diagnosis. Patients were stratified into surgery and biopsy groups, respectively, according to their treatment. There were significant differences between the two groups in terms of clinical factors, including symptoms (*P* = .017), number of lesions (*P* < .001), chemo‐ or radio‐therapy (*P* < .001), and stages (*P* = .003).

**Table 1:** Demographic characteristics according to treatment with or without surgical resection

Characteristics	No.	Surgery	Biopsy	*P*
Age		58.5 ± 9.43	58.6 ± 11.55	.968
≤60	46	18	28	.726
>60	34	12	22	
Gender				.073
Male	37	10	27	
Female	43	20	23	
Symptom				.017
Absent	27	15	12	
Present	53	15	38	
Symptom B				.057
Absent	63	27	36	
Present	17	3	14	
No. of lesions				<.001
1	36	25	11	
2	11	4	7	
3 or more	33	1	32	
Stage				.003
I	56	27	29	
II	14	3	11	
III	3	0	3	
IV	7	0	7	
Procedure of diagnosis				<.001
Bronchoscopy biopsy	33	3	30	
Bercutaneous biopsy	14	0	14	
Wedge resection	14	8	6	
Segmentectomy or lobectomy	19	19	0	
Chemo‐ or radio‐therapy				<.001
Absent	41	23	18	
Present	39	7	32	

**Figure 2:**
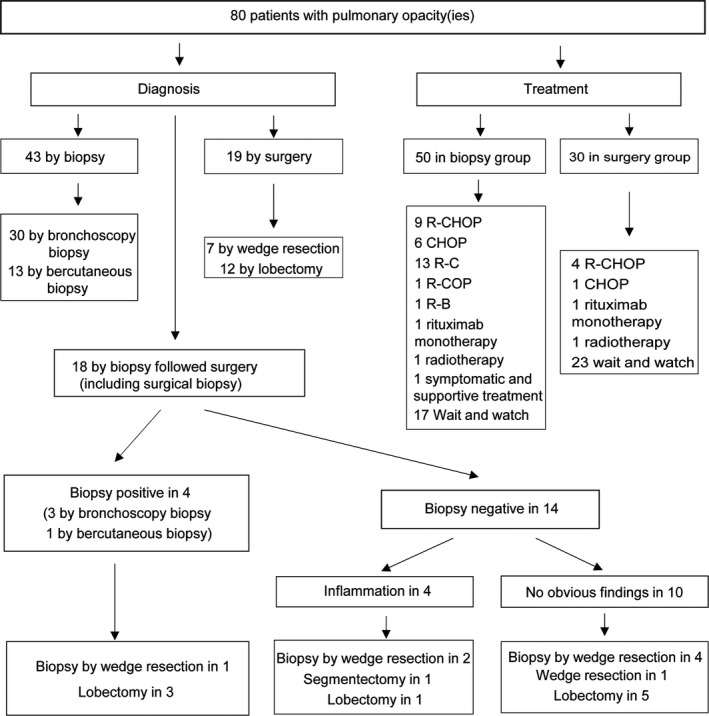
Strategy for pathological diagnosis and treatment in pulmonary MALT lymphoma

Thirty patients were treated with surgery. Lobectomy was the most frequent procedure and was conducted in 66.6% of all surgical patients. Wedge resection was conducted in eight patients and segmentectomy in the other two. Six patients received postoperative adjuvant chemotherapy (R (rituximab)‐CHOP (cyclophosphamide, doxorubicin, vincristine, and prednisone) in four patients, CHOP in one patient, and rituximab monotherapy in one patient) and one patient received involved‐field radiotherapy. Twenty‐three patients did not receive postoperative adjuvant therapy. Thirty‐one patients were treated with chemotherapy alone, including R‐C (cladribine) in 13 patients, CHOP in six patients, R‐CHOP in nine patients, and R‐COP (cyclophosphamide, vincristine, and prednisone), R‐B (Chlorambucil), and rituximab monotherapy in one patient each. One patient was treated with radiotherapy and one received only symptomatic and supportive treatment. Waiting and watching without treatment was recommended in 17 patients. The diagnostic procedures and treatment modalities were provided in Figure 2.

**Figure 3:**
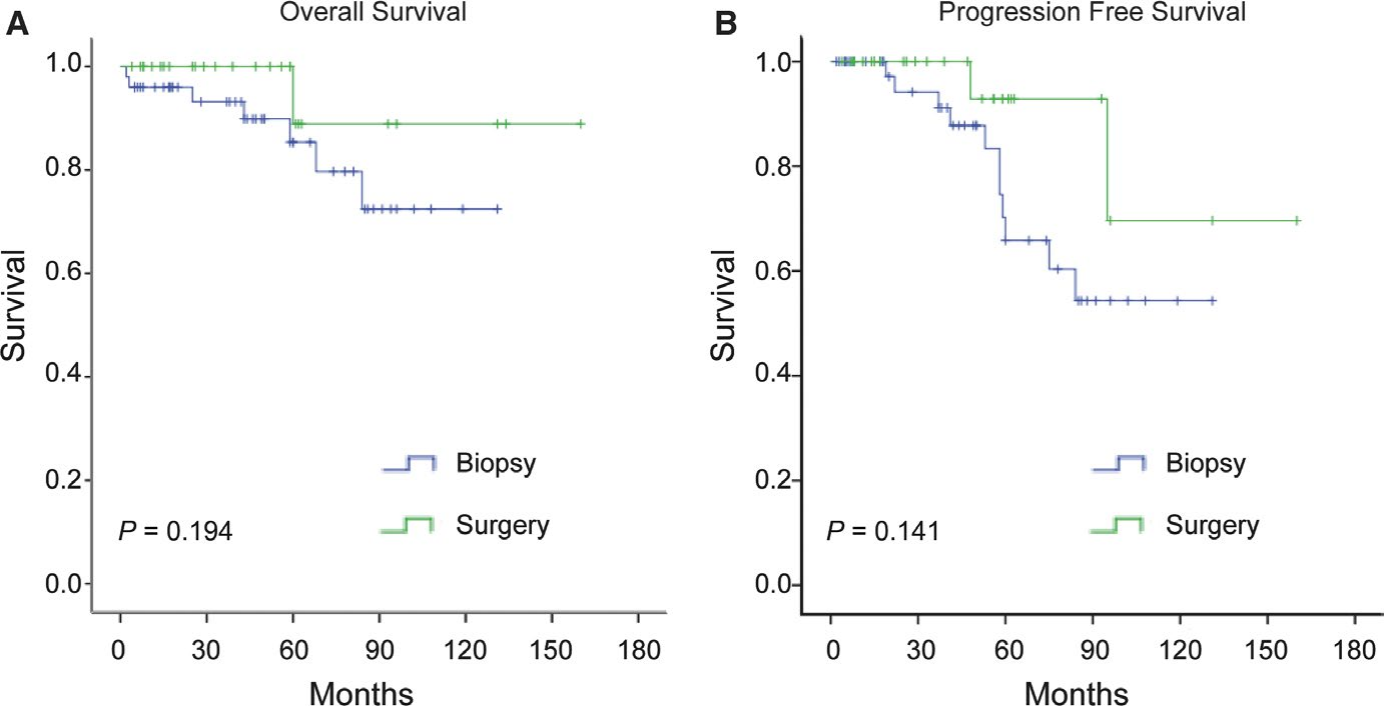
Kaplan‐Meier curves comparing overall survival (A) and progression‐free survival (B) between biopsy and surgery groups in pulmonary MALT lymphoma patients

13 of 80 (16.25%) patients experienced progression/relapse during the period of observation. The estimated 5‐year PFS rate was 74.6% in our study cohort. The comparison of PFS between surgery and biopsy groups was shown in Figure 3. Although PFS analysis showed a trend in favor of patients treated with surgery, there was no significant difference. Factors associated with a higher probability of shorter PFS are shown in Table 2. The number of lesions was associated with a higher probability of shorter PFS in the univariate analysis, and was confirmed in multivariate analysis. Neither age, gender, symptom, surgery, chemo‐, or radio‐therapy, stage were predictors of PFS.

**Table 2:** Univariate and multivariate analysis of progression‐free survival

Variable	Univariate			Multivariate		
	HR	95% CI	*P*	HR	95% CI	*P*
Age (1 year increasing)	1.027	0.976‐1.080	.312			
(>60 vs ≤60)	2.324	0.751‐7.191	.143			
Gender (Male vs female)	1.727	0.561‐5.313	.341			
Symptom (Present vs absent)	2.148	0.474‐9.729	.321			
Symptom B (Present vs absent)	0.698	0.190‐2.566	.588			
No. of lesions (1 level increasing)	2.958	1.330‐6.580	.008	3.614	1.094‐11.942	.035
Therapy (Surgery vs biopsy)	0.322	0.071‐1.455	.141	0.867	0.271‐2.776	.810
Chemo‐ or radio‐therapy (Present vs absent)	1.339	0.432‐4.150	.613	1.666	0.181‐15.350	.652
Stage (II, III, IV vs I)	0.909	0.295‐2.799	.868			

**Figure 4:**
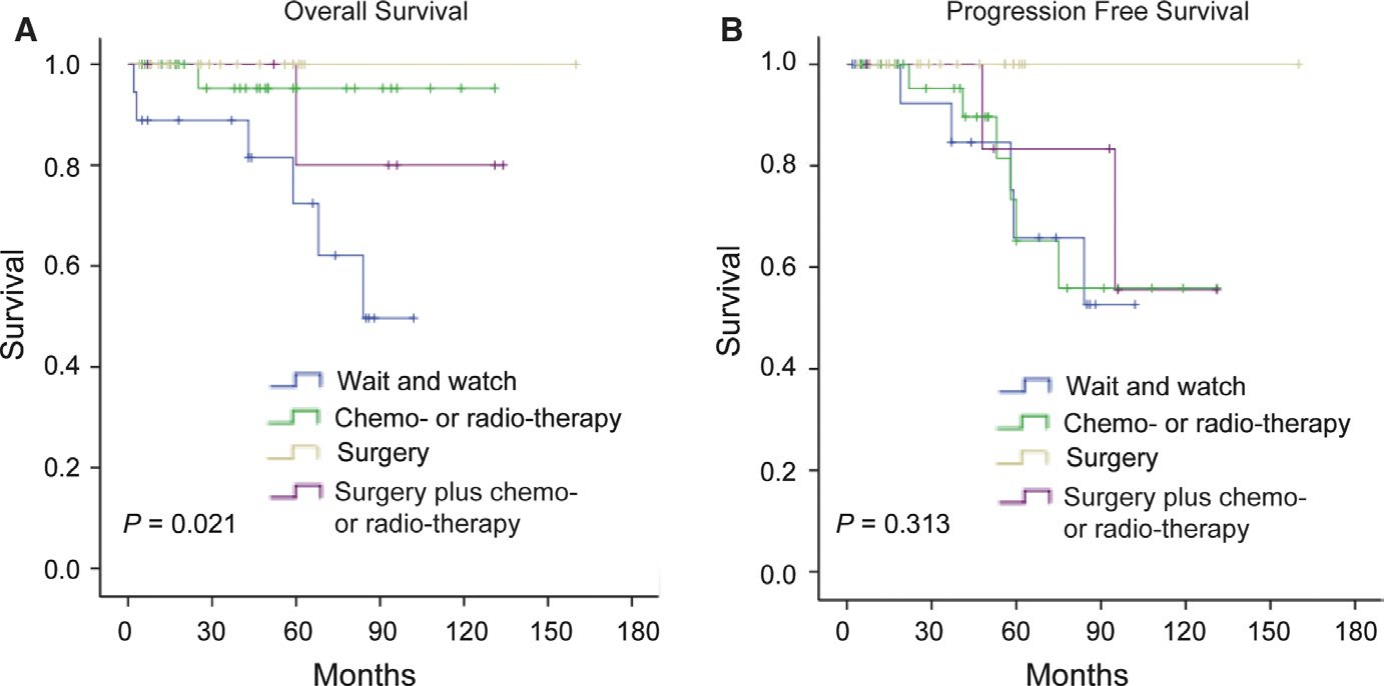
Kaplan‐Meier curves comparing overall survival (A) and progression‐free survival (B) between different treatment strategies in pulmonary MALT lymphoma patients

**Figure 5:**
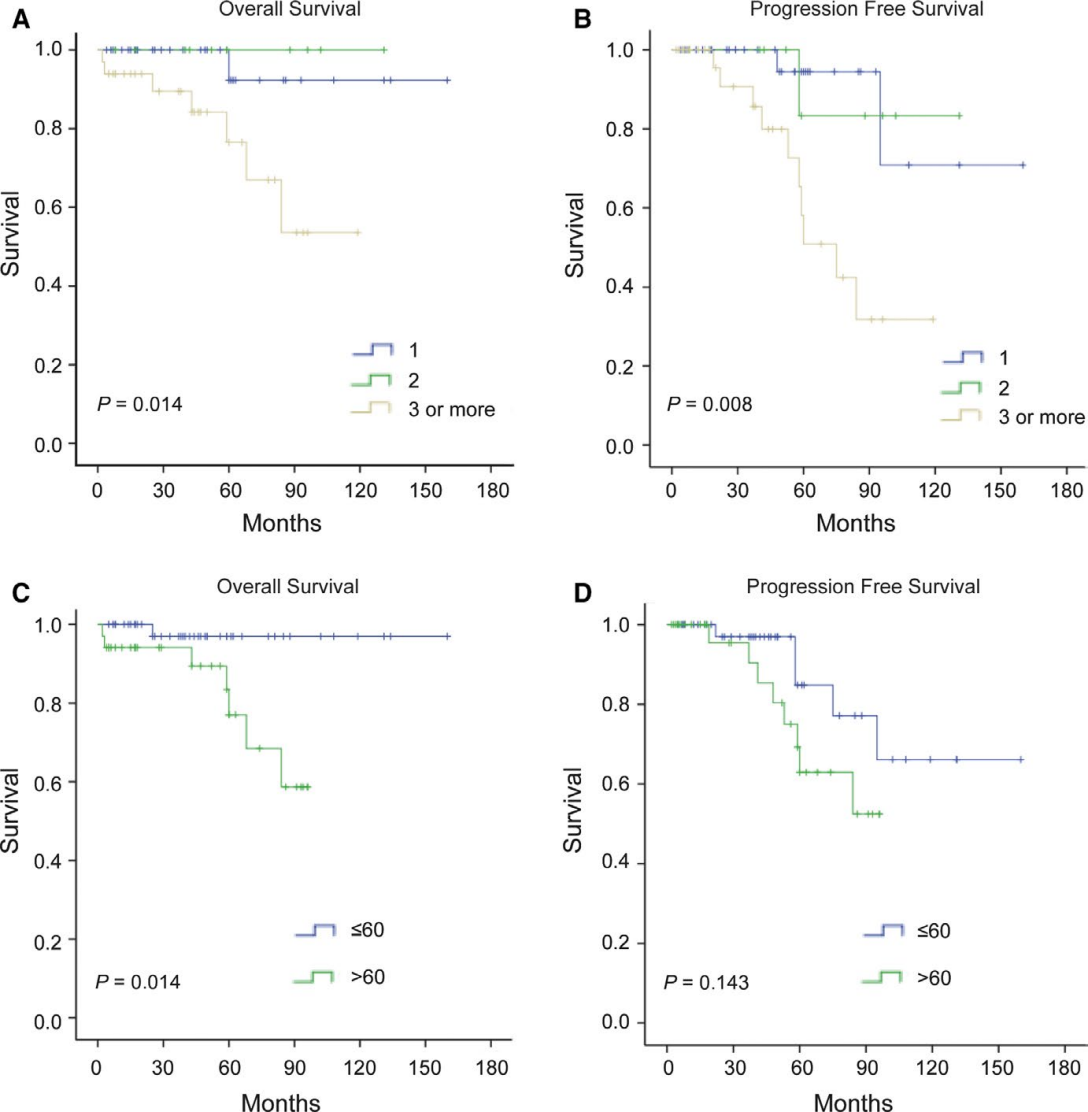
Kaplan‐Meier curves comparing overall survival (A) and progression‐free survival (B) between different number of lesions in pulmonary MALT lymphoma patients. Kaplan‐Meier curves comparing overall survival (C) and progression‐free survival (D) different age groups in pulmonary MALT lymphoma patients

Eight patients (10.0%) died during the follow‐up period. The estimated 5‐year OS rate was 87.1%. There was no significant difference in OS between patients treated with and without surgical resection (Figure 3). In the prognoses analyses combined surgery and chemo‐ or radio‐therapy, there was significant difference in OS (Figure 4). Log‐rank analysis identified number of lesions and age (Figure 5) as factors associated with OS. Multivariate analysis including number of lesions, age, chemo‐, or radio‐therapy and surgery identified age as the only independent prognostic factor (Table 3).

**Table 3:** Univariate and multivariate analysis of overall survival

Variable	Univariate			Multivariate		
	HR	95% CI	*P*	HR	95% CI	*P*
Age (1 year increasing) (>60 vs ≤60)	1.155 8.778	1.049‐1.273 1.078‐71.452	.001 .014	1.164	1.019‐1.331	.025
Gender (Male vs female)	2.956	0.594‐14.709	.165			
Symptom (Present vs absent)	34.188	0.044‐26818.762	.083			
Symptom B (Present vs absent)	0.351	0.042‐2.897	.310			
No. of lesions (1 level increasing)	3.660	1.099‐12.188	.014	5.081	0.705‐36.628	.107
Therapy (Surgery vs biopsy)	0.273	0.033‐2.226	.194	3.681	0.089‐151.739	.492
Chemo‐ or radio‐therapy (Present vs absent)	0286	0.057‐1.427	.104	0.080	3.315	.485
Stage (II, III, IV vs I)	1.580	0.389‐6.423	.519			

## 4 DISCUSSION

Many MALT lymphoma patients are asymptomatic (33.8% in this study), and even when symptoms are present, they are usually nonspecific pulmonary symptoms such as cough, dyspnea, chest pain, fatigue, and chest tightness. Night sweats, weight loss, and fever were also present in 22.3% of patients in this study. The current findings were consistent with previous studies, which indicated that respiratory symptoms presented in 55%‐63% patients and B symptom presented in 15%‐22% patients.^1^, ^13^, ^15^, ^16^ Similar to the symptoms, the imaging findings of MALT lymphoma are also nonspecific. Atelectasis, masses, nodules, ground glass opacity changes, and pleural effusion were frequently observed in our study, making it difficult to differentiate MALT lymphoma from lung cancer and pneumonia. Its nonspecific symptoms and imaging findings thus make the diagnosis of MALT lymphoma difficult.

Pulmonary MALT lymphoma can only be reliably diagnosed by invasive pathologic methods, such as surgical resection, transbronchial lung biopsy, or CT‐guided transthoracic lung biopsy to obtain samples for pathologic diagnosis. However, a previous study suggested that bronchofiberoscopy may be of limited diagnostic value,^17^ whereas another study reported that the positive predictive rate of puncture biopsy under CT guidance for diagnosing pulmonary non‐Hodgkin's lymphoma was only 25%.^18^ A further study found that minimally invasive approaches including CT‐guided percutaneous transthoracic biopsies, fiberoptic bronchoscopy and transbronchial biopsies were the most frequently used method for diagnosis and more than 70% patients were diagnosed in this way.^13^ Given that multi‐lobar lesions or disseminated disease appeared in most patients, these minimal diagnostic approaches seem justified. Although most patients in this study were diagnosed by minimally invasive procedures, a single minimally invasive procedure was not sufficient to make an accurate diagnosis. Bronchoscopy or bercutaneous biopsy were performed in 18 patients who underwent surgery but had a low diagnostic yield, with nine patients showing no obvious abnormalities and four diagnosed with inflammatory lesions; only four patients were confirmed with MALT lymphoma because of the infrequency of endoluminal lesions.

In our experience, minimally invasive biopsy was insufficient to obtain a definitive diagnosis. Too little tissue was obtained by biopsy, and it was difficult to determine if it indicated reactive hyperplasia or neoplastic hyperplasia. Several non‐neoplastic reactive conditions can simulate lymphoma and diseases with reactive lymphoid proliferations are morphologically difficult to distinguish from primary malignant lymphoid tumors.^19^ Needle biopsy samples, even when they are large enough for histological analysis, have the disadvantage that the obtained tissue is not necessarily representative; notably, it is more difficult to distinguish between tumor cells and cells from the host response surrounding the tumor in lymphomas compared with epithelial neoplasms. Furthermore, secondary pulmonary parenchymal changes and secondary changes in the lymphoma itself, such as necrosis, may be more extensive than in unchanged areas. This is an important reason to obtain sufficient biopsy material for an accurate diagnosis of this disease.^3^ Overall, these results indicate that biopsy has a low diagnostic value for local lesions, and may result in a high rate of missed diagnoses. A more generous surgical intervention, involving thoracotomy or video‐assisted thoracoscopic surgery, should thus, be considered to make a diagnosis in most patients with a primary pulmonary lymphoma, in order to reduce the misdiagnosis rate.

Surgery can provide diagnostic material and achieve therapeutic resection in the management of MALT lymphomas. However, the optimal management of MALT lymphoma remains unclear, as reflected by the different approaches used in the past. However, pulmonary MALT lymphomas have a satisfactory survival outcome irrespective of the therapeutic strategy, including waiting and watching, chemotherapy, or surgery. MALT lymphoma is a rare malignant tumor and no evidence of prospective researches are currently available, and therapeutic protocols are therefore mainly on the basis of expert opinion. Several retrospective studies have indicated the effectiveness and safety of chemotherapy and rituximab monotherapy in patients with proven MALT lymphoma.^20^, ^21^ However, surgery has been recommended in cases of localized MALT lymphoma when complete resection can be achieved,^16^ and selected patients receiving surgical resection had better 5‐year progression‐free survival than those who received chemotherapy.^15^ Importantly, most patients with MALT lymphoma stage IE (nodal negative) who received surgical resection had relatively longer survival compared with those without surgery, regardless of whether they had received chemotherapy.^22^ Another study suggested that surgical resection might not be the first choice for therapy in pulmonary MALT lymphoma patients for whom surgery is not necessary for diagnosis, even if the lesions are localized, considering preservation of lung function and avoidance of surgical risks. One study revealed that there was no difference between surgery and chemotherapy in terms of progression and survival, due to the favorable clinical course of MALT lymphoma itself.^23^ Although the surgery group had a better prognosis than the biopsy group in this study, the difference was not significant. However, the distribution of patients in the surgery and biopsy groups was significantly different in terms of tumor number and stage, making it difficult to evaluate the role of surgery in MALT lymphoma.

Pulmonary MALT lymphoma usually has an indolent course, remaining localized in the lung for long periods before dissemination. These tumors, therefore, respond well to local or systemic therapy and have a relatively good prognosis.^24^ This study confirmed the favorable course of pulmonary MALT lymphoma, with an estimated 87.1% 5‐year survival rate. However, despite its favorable prognosis, several factors have been associated with its prognosis in previous studies, including the presence of B symptoms, tumor microvascular density, and elevated lactate dehydrogenase serum levels,^22^ age and performance status,^13^ lymph node and extrapulmonary metastases,^23^ and modified Ann Arbor staging.^16^ Since only eight patients died in our study, only age, tumor number, and treatment approach were the prognostic factors associated with survival. Waiting and watching has become an important approach in the management of MALT lymphoma, because of its indolent nature. Troch et al conducted a study in which patients with pulmonary MALT lymphoma were merely followed up without treatment. They pointed out that pulmonary MALT lymphoma was a very indolent disease with the potential for spontaneous regression, and that asymptomatic patients might not require immediate treatment.^25^ However, the patient without treatment in this study had a poorer OS than patients who received chemotherapy, suggesting that a wait and watch approach should be used with caution and only in highly selected patients. Overall, age was identified as the only independent prognostic factor, possibly reflecting the indolent nature of MALT lymphoma and its favorable prognosis. Considering the overall survival may be confounded by patients’ underlying disease, PFS, reflecting tumor growth in indolent disease, is probably more useful for prediction of treatment efficacy than OS. The estimated 5‐year PFS rate was 74.6% in our study. Although PFS analysis showed a trend in favor of patients treated with surgery, we cannot conclude that surgery is beneficial to PFS because of the less number of tumors and earlier stages in the surgery group. Meanwhile, no correlation was found between wait and watch approach and worse PFS of pulmonary MALT patients. Univariate and Multivariate analysis showed the only factor associated with shorter PFS was the number of lesions. In our study, more pulmonary lesions were also associated with shorter OS, although it was not an independent factor in multivariate analysis. The number of lesions was related to bilateral lung involvement, and a previous study suggested that bilateral lung involvement was associated worse OS, but there was no significant difference in PFS.^23^ The number of lesions may reflect the tumor burden, and our research suggested that it was the most important prognostic factor not only in PFS but also in OS analysis.

## 5 CONCLUSION

In conclusion, our findings confirmed that pulmonary MALT lymphoma is an indolent disease with favorable treatment outcomes. In line with its indolent nature, age was the only independent prognostic factor for OS in patients with MALT lymphoma. Surgery still plays an important role in the diagnosis and treatment of MALT lymphoma. However, larger scale studies are needed to better evaluate the optimal treatment for patients with pulmonary MALT lymphoma.

## CONFLICT OF INTEREST

There are no conflicts of interest to declare.

## References

[1] Cardenas‐Garcia J , Talwar A , Shah R , Fein A . Update in primary pulmonary lymphomas. Curr Opin Pulm Med. 2015;21(4):333‐337. 25978630 10.1097/MCP.0000000000000180

[2] Ferraro P , Trastek VF , Adlakha H , et al. Primary non‐Hodgkin's lymphoma of the lung. Ann Thorac Surg. 2000;69(4):993‐997. 10800781 10.1016/s0003-4975(99)01535-0

[3] Habicht JM , Lori A , Stulz P , et al. Primary non‐Hodgkin's lymphoma of the lung. Will videothoracoscopic biopsy change decision‐making with regard to resection of this disease? Thorac Cardiovasc Surg. 1994;42(2):112‐115. 8016825 10.1055/s-2007-1016468

[4] McCulloch GL , Sinnatamby R , Stewart S , Goddard M , Flower CD . High‐resolution computed tomographic appearance of MALToma of the lung. Eur Radiol. 1998;8(9):1669‐1673. 9866784 10.1007/s003300050609

[5] Parsonnet J , Hansen S , Rodriguez L , et al. Helicobacter pylori infection and gastric lymphoma. N Engl J Med. 1994;330(18):1267‐1271. 8145781 10.1056/NEJM199405053301803

[6] Borie R , Wislez M , Antoine M , et al. Pulmonary mucosa‐associated lymphoid tissue lymphoma revisited. Eur Respir J. 2016;47(4):1244‐1260. 26797028 10.1183/13993003.01701-2015

[7] Ferreri AJ , Guidoboni M , Ponzoni M , et al. Evidence for an association between *Chlamydia psittaci* and ocular adnexal lymphomas. J Natl Cancer Inst. 2004;96(8):586‐594. 15100336 10.1093/jnci/djh102

[8] Ascoli V , Lo CF , Artini M , et al. Extranodal lymphomas associated with hepatitis C virus infection. Am J Clin Pathol. 1998;109(5):600‐609. 9576580 10.1093/ajcp/109.5.600

[9] Cerroni L , Zochling N , Putz B , Kerl H . Infection by *Borrelia burgdorferi* and cutaneous B‐cell lymphoma. J Cutan Pathol. 1997;24(8):457‐461. 9331890 10.1111/j.1600-0560.1997.tb01318.x

[10] Chanudet E , Adam P , Nicholson AG , et al. Chlamydiae and Mycoplasma infections in pulmonary MALT lymphoma. Br J Cancer. 2007;97(7):949‐951. 17876330 10.1038/sj.bjc.6603981 PMC2360427

[11] Adam P , Czapiewski P , Colak S , et al. Prevalence of *Achromobacter xylosoxidans* in pulmonary mucosa‐associated lymphoid tissue lymphoma in different regions of Europe. Br J Haematol. 2014;164(6):804‐810. 24372375 10.1111/bjh.12703

[12] Aoyama S , Masaki A , Sakamoto Y , et al. Achromobacter infection is rare in Japanese patients with pulmonary B‐cell lymphoma. Intern Med. 2018;57(6):789‐794. 29151525 10.2169/internalmedicine.9430-17 PMC5891515

[13] Borie R , Wislez M , Thabut G , et al. Clinical characteristics and prognostic factors of pulmonary MALT lymphoma. Eur Respir J. 2009;34(6):1408‐1416. 19541720 10.1183/09031936.00039309

[14] Cook JR , Isaacson PG , Chott A , et al. Extranodal marginal zone lymphoma of mucosa‐associated lymphoid tissue (MALT lymphoma) In: Swerdlow SH, Campo E, Harris NL, Jaffe ES, Pileri SA, Stein H, Jurgen T, eds. WHO Classification of Tumours of Haematopoietic and Lymphoid Tissues. Revised Fourth Edition. Lyon: IARC 2017;259‐262.

[15] Sammassimo S , Pruneri G , Andreola G , et al. A retrospective international study on primary extranodal marginal zone lymphoma of the lung (BALT lymphoma) on behalf of International Extranodal Lymphoma Study Group (IELSG). Hematol Oncol. 2016;34(4):177‐183. 26152851 10.1002/hon.2243

[16] Lee H , Yang B , Nam B , et al. Treatment outcomes in patients with extranodal marginal zone B‐cell lymphoma of the lung. J Thorac Cardiovasc Surg. 2017;154(1):342‐349. 28457544 10.1016/j.jtcvs.2017.03.043

[17] Xu HY , Jin T , Li RY , et al. Diagnosis and treatment of pulmonary mucosa‐associated lymphoid tissue lymphoma. Chin Med J (Engl). 2007;120(8):648‐651. 17517178

[18] Graham BB , Mathisen DJ , Mark EJ , Takvorian RW . Primary pulmonary lymphoma. Ann Thorac Surg. 2005;80(4):1248‐1253. 16181848 10.1016/j.athoracsur.2005.04.014

[19] Nicholson AG , Wotherspoon AC , Diss TC , et al. Reactive pulmonary lymphoid disorders. Histopathology. 1995;26(5):405‐412. 7544761 10.1111/j.1365-2559.1995.tb00247.x

[20] Zinzani PL , Pellegrini C , Gandolfi L , et al. Extranodal marginal zone B‐cell lymphoma of the lung: experience with fludarabine and mitoxantrone‐containing regimens. Hematol Oncol. 2013;31(4):183‐188. 23212941 10.1002/hon.2039

[21] Okamura I , Imai H , Mori K , et al. Rituximab monotherapy as a first‐line treatment for pulmonary mucosa‐associated lymphoid tissue lymphoma. Int J Hematol. 2015;101(1):46‐51. 25378228 10.1007/s12185-014-1694-8

[22] Yang M , Zhao S , Zhang X , et al. Effects of microvascular density on primary pulmonary non‐Hodgkin's lymphoma (PPL). Tumour Biol. 2012;33(6):2143‐2150. 22875783 10.1007/s13277-012-0474-4

[23] Oh SY , Kim WS , Kim JS , et al. Pulmonary marginal zone B‐cell lymphoma of MALT type–what is a prognostic factor and which is the optimal treatment, operation, or chemotherapy?: Consortium for Improving Survival of Lymphoma (CISL) study. Ann Hematol. 2010;89(6):563‐568. 20024551 10.1007/s00277-009-0875-7

[24] Ahmed S , Kussick SJ , Siddiqui AK , et al. Bronchial‐associated lymphoid tissue lymphoma: a clinical study of a rare disease. Eur J Cancer. 2004;40(9):1320‐1326. 15177490 10.1016/j.ejca.2004.02.006

[25] Troch M , Streubel B , Petkov V , et al. Does MALT lymphoma of the lung require immediate treatment? An analysis of 11 untreated cases with long‐term follow‐up. Anticancer Res. 2007;27(5B):3633‐3637. 17972528

